# Body composition and chemotherapy toxicities in breast cancer: a systematic review of the literature

**DOI:** 10.1007/s11764-023-01512-z

**Published:** 2024-01-11

**Authors:** Lori Lewis, Belinda Thompson, Rhiannon Stellmaker, Louise Koelmeyer

**Affiliations:** Australian Lymphoedema Education, Research & Treatment (ALERT) Program, Department of Health Sciences, Faculty of Medicine, Health and Human Sciences, Macquarie University, Level 1, 75 Talavera Road, Sydney, NSW 2109 Australia

**Keywords:** Breast cancer, Chemotherapy, Toxicities, Sarcopenia

## Abstract

**Purpose:**

Breast cancer is the most diagnosed cancer in women with chemotherapy being a common treatment. Toxicities due to chemotherapy can result in dose reduction, delay, and early cessation of treatment, which along with causing distress for individuals during their cancer treatment might also reduce the therapeutic effect. The purpose of this systematic review is to examine the role of body composition on chemotherapy toxicities in women with breast cancer.

**Methods:**

A systematic search of the literature was completed on electronic databases Pubmed, Embase, CINHAHL, and Cochrane. Studies were included if the direct effect of body composition on chemotherapy toxicities was reported and excluded if body composition could not be isolated. A critical appraisal of the studies included was performed using McMasters University Critical Review Form for Quantitative Studies.

**Results:**

Eleven studies were included with a total of 2881 female participants. All studies reported significant relationships between body composition and chemotherapy toxicities; however, individual parameters differed between the studies. Adding to the heterogeneity, different thresholds were reported to determine both sarcopenia and myosteatosis, making it difficult to identify a common finding.

**Conclusion:**

This review suggests that body composition may be an important factor in predicting the severity of chemotherapy toxicities during treatment for breast cancer; however, the lack of international consensus as to thresholds in the literature for sarcopenia and myosteatosis may result in bias. The review supports the need for further prospective studies, allowing for more robust, pre-determined data collection, to better understand the implications of body composition on toxicities and benefits of using body composition to individualize chemotherapy dosing.

**Implications for Cancer Survivors:**

Toxicities due to chemotherapy can result in treatment being unable to be completed as planned, potentially resulting in poorer survival outcomes. Improved knowledge in this area may give rise to a more reliable way of individualizing chemotherapy dosage to help mitigate this risk.

**Supplementary Information:**

The online version contains supplementary material available at 10.1007/s11764-023-01512-z.

## Introduction

Breast cancer is the most frequently diagnosed cancer and the primary cause of cancer deaths in women worldwide [1, 2], with approximately 2.26 million cases and 685 000 deaths reported in 2020 [2, 3]. The goal of treatment for early-stage breast cancer is to eliminate the primary tumor/s and prevent the development of metastatic disease. For metastatic disease, which in most cases is incurable, therapy aims to reduce and palliate symptoms and prolong life expectancy [4, 5]. Different combinations of multimodality treatments both local (surgery and radiation) and systemic (chemotherapy and endocrine therapy) are used for the management of breast cancer. Chemotherapy is a commonly used systemic treatment that can be administered both pre (neo-adjuvant) and post (adjuvant) surgery. Chemotherapy has been shown to reduce the risk of distant metastases and has had a considerable impact on overall breast cancer prognosis and survival [6, 7]. Toxicities due to chemotherapy can be challenging with hematologic (neutropenia; febrile neutropenia), gastrointestinal (nausea; diarrhea), and neurological (peripheral neuropathy) symptoms being commonly observed [8]. In addition to causing individuals distress, these toxicities may also result in chemotherapy treatment being delayed or even ceased early, possibly reducing the therapeutic effect.

The ability of individuals to metabolize and eliminate drugs from the body can vary considerably [9, 10]. Body surface area (BSA) calculated using height and weight is often used to calculate the dose of cytotoxic chemotherapy drugs. This method was adopted in the 1950s [11, 12], however, has recently been questioned as it does not consider the disparity in an individual’s pharmacokinetics [13, 14] or body composition. Cytotoxic drugs are known to have a narrow therapeutic window meaning that even small changes in dose can have a significant effect. If the dose is too high, it may lead to increased severity of toxicities, and if too low may result in sub-optimal outcomes due to the reduced therapeutic effect [9, 10, 12, 15]. As toxicity from cytotoxic chemotherapy drugs is expected, the ideal dose would result in the greatest tumor response along with manageable levels of toxicity [12].

In recent years, there has been increasing interest in body composition, in particular sarcopenia and muscle mass, as a predicting factor in both cancer prognosis and adverse effects during treatment [13, 16, 17]. Sarcopenia was first described in the 1980s as a decrease in lean muscle mass associated with aging, affecting mobility and independence. This definition has since been expanded to incorporate function and muscle strength in conjunction with low skeletal muscle mass and is no longer linked exclusively to aging [18, 19]. Although sarcopenia is commonly described and diagnosed, and consensus guidelines exist [20, 21], there is no internationally accepted definition of the exact parameters used to make a diagnosis of sarcopenia [18, 20–22]. Additionally, no clearly defined screening tool has been identified to aid with diagnosis [22]. As body composition can vary considerably between individuals with similar BSA, this leads to the question of the validity of BSA as an accurate means for calculating chemotherapy dose. Given the growing evidence that lean body mass may better predict drug distribution and clearance [10, 12, 16, 23, 24], going forward, this may be a more reliable parameter for dose calculation. A systematic review completed in 2019 found that sarcopenia (low skeletal muscle index) along with low muscle density was significantly associated with a higher risk of grade 3–5 chemotherapy toxicities (according to National Cancer Institute Common Toxicity Criteria for Adverse Events (NCI-CTCAE; Version 4.03)) in both early and metastatic breast cancer [25]; however, toxicities were the secondary outcome in this review with only three studies included in the analyses. Additional research into other cancer groups has also demonstrated that individuals with sarcopenia have greater toxicities from cytotoxic chemotherapy, often resulting in the delay or early cessation of treatment [13, 26, 27]. This in turn may lead to poorer outcomes brought about by decreased dose, resulting in a reduced therapeutic effect [28]. Current literature supports further investigation into the potential role of routine body composition assessments as a means of calculating accurate chemotherapy dosage for individuals undergoing treatment for all cancer types.

There are various means of calculating body composition such as magnetic resonance imaging (MRI), computerized tomography (CT), dual-energy X-ray absorptiometry (DEXA), bioelectrical impedance analysis (BIA), and bioimpedance spectroscopy (BIS). Each of these methods have advantages and limitations. Both MRI and CT use cross-sectional analysis to quantify the volume and characteristics of skeletal muscles along with visceral and subcutaneous adipose tissue. MRI and CT are both expensive and time-consuming; however, for individuals with cancer, both MRI and more commonly CT are routinely performed to assess cancer staging. A benefit of MRI is that it does not expose the individual to radiation; however, a limitation is that it is less commonly used for cancer staging. An advantage of CT is that although it exposes the individual to radiation, it is a commonly used imaging technique to assess cancer staging. It is also highly accurate and able to quantify lean body mass, and both visceral and subcutaneous fat; however, it is unable to assess muscle quality [29, 30]. Analysis of body composition using CT requires specialized software for analysis [29, 30] and is often performed using the axial image at the level of the third lumbar vertebrae. Muscle area along with visceral, subcutaneous, and intramuscular adipose tissue identified from a cross-sectional CT slice at the level of L3 has been shown to be directly associated with that of the whole body [31] and is therefore commonly used to assess body composition [29, 32].

DEXA is a full-body imaging scan that uses X-rays to evaluate lean body mass (LBM), fat mass (FM), and bone mineral mass (BMM). DEXA is considered the gold standard for bone mineral density; however, as it is a two-dimensional scan, it is unable to distinguish between visceral and subcutaneous fat [33]. The advantages of DEXA are that it only exposes individuals to low-dose radiation compared to CT, and it has been shown to be accurate and reproducible [30, 34]. However, DEXA requires expensive, specialized equipment and a trained technician to perform the scan [34]. It is also unable to distinguish between types of lean soft tissue; therefore, increased fluid levels can result in the assumption of higher muscle mass. More recently, BIA and BIS have been used as a means of assessing body composition by analyzing the impedance or opposition to a low, multi-frequency electrical current as it is passed through the body to assess parameters such as FM, fat-free mass (FFM), skeletal muscle mass (SMM), and hydration levels. The advantage of these methods is that there is no exposure to radiation, and they can be easily performed in a clinical setting [29, 34]. Disadvantages are that BIS tends to underestimate FFM when compared to DEXA in people of normal weight and overestimate in those who are obese [34].

With growing evidence supporting the theory that body composition parameters can be predictors of both prognosis after cancer diagnosis and the risk of increased chemotherapy toxicities, it stands to reason that implementing body composition assessment as part of routine care may improve clinical outcomes for individuals undergoing treatment for cancer. The aim of this systematic review is to evaluate current literature examining the role of body composition on toxicities experienced during chemotherapy treatment for breast cancer, to guide the direction for future studies into this under-researched area.

## Methods

### Data sources and searches

An electronic database search of titles and abstracts in PubMed, Cochrane, CINAHL, and Embase was completed up to the 24th of August 2022. The search term breast cancer was combined with chemotherapy, cytotoxic, toxicity, side effects, body composition, sarcopenia, muscle mass, fat mass, cachexia, malnutrition, undernutrition, sarcopenic obesity, and anorexia (Supplementary File 1). Due to recent advances in chemotherapy regimens and body composition analyses, the search was restricted to studies from 2012 onwards. Studies included were limited to full text, English language, and humans; however, there was no restriction on study type. Additionally, all included studies were manually screened by LL and RS.

### Study inclusion

The inclusion criteria were women over the age of 18, who were undergoing chemotherapy (any regimen) for any type or stage of breast cancer. Published studies were included if the specific effect of body composition on the severity of chemotherapy toxicities was analyzed. Body composition parameters must at a minimum include muscle mass, fat mass, or fat-free mass in addition to body mass index (BMI) and/or body surface area (BSA). The outcome measure for included studies were chemotherapy toxicities and included both graded toxicities reduced dose, and/or delay of, or early cessation of, treatment. Studies were excluded if the effect of body composition could not be isolated due to additional interventions.

### Data extraction and quality assessment

Electronic data searches were performed by one reviewer (LL) after which titles and abstracts were evaluated independently by 2 reviewers (LL, RS). Articles were excluded if the titles or abstracts clearly did not meet the inclusion criteria. Discrepancies regarding studies were discussed by both reviewers and if a consensus could not be achieved a third reviewer (BT) assisted to resolve the issue. A standardized data extraction form was used to obtain information on participant characteristics, study inclusion/exclusion criteria, assessments performed, outcome data analyzed, and study conclusions on each of the included articles.

The McMaster University Guidelines and Critical Review Form for Quantitative Studies was utilized to critically assess the methodological quality of included studies [35]. The McMaster critical review for quantitative studies is a numerical scoring system ranging from 0 to 15 with a score of 1 point for yes answers and 0 for no answers over a variety of categories. In previous systematic reviews, scores ranging from seven to nine have been interpreted to indicate moderate quality, and scores of greater than or equal to ten, high-quality methodological studies [36, 37]. All studies incorporated in the review used quantitative methodological models; therefore, this guideline was deemed an appropriate analysis tool. The assessments were performed by two independent reviewers (LL and RS) after which scores were compared and evaluated by a third reviewer (BT) if any discrepancies were noted. The third reviewer was required for two of the 11 included articles.

## Results

### Study selection

**Fig. 1:**
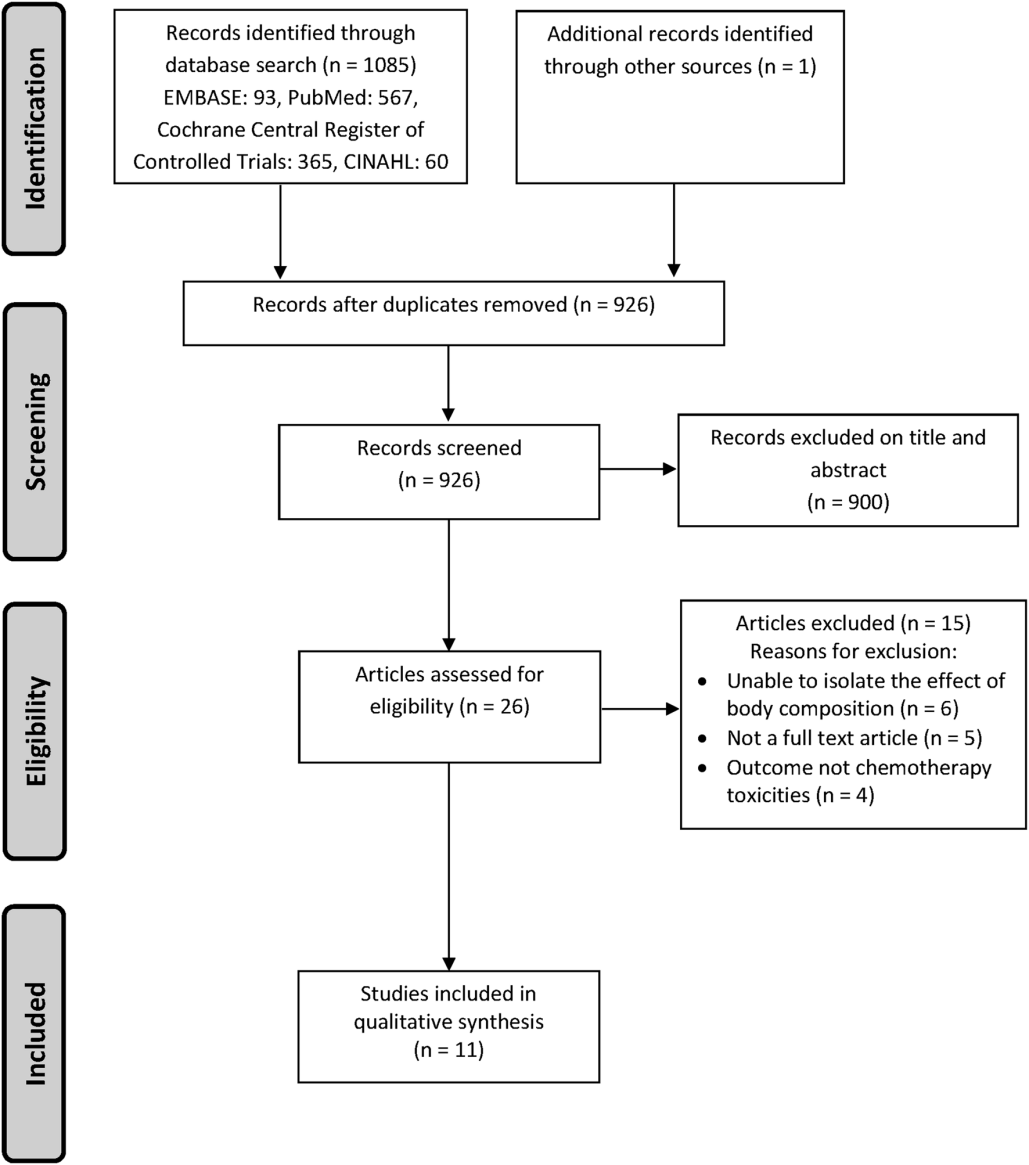
Flowchart showing the screening process and search results

The initial database search generated a total of 1085 records, with 926 remaining after duplicates were removed. Of the 926 articles screened, a total of 26 were considered potentially eligible after reviewing titles and abstracts. Following a full-text review of the remaining 26 articles, 15 were excluded (Supplementary File 2) and 11 were accepted for inclusion in the systematic review (Fig. 1).

### Methodological quality

Studies included in the review were evaluated for quality using the McMasters methodological quality assessments. Scores ranged from seven to 13, with eight of the 11 studies scoring 10 or higher showing high methodological quality, and the remaining three moderate methodological quality (Table 1). Only one of the 11 studies justified their sample size [40] and four did not address either contamination or co-intervention [17, 38, 41, 42]. Nine of the 11 studies were retrospective [17, 38, 39, 41–46], and none were randomized, controlled studies.

**Table 1:** Critical review (possible scores are shown in brackets)

Author(s)	Study purpose (1)	Literature (1)	Design (1)	Sampling (2)	Outcomes (2)	Intervention (3)	Results (4)	Conclusions (1)	Score (_/15)	Quality
Aleixo et al. (2021)	1	1	1	1	0	2	4	1	11	High
Aleixo et al. (2020)	1	1	1	1	1	2	4	0	11	High
Cespedes et al. (2019)	1	1	1	1	0	1	4	1	10	High
Gouérant et al. (2013)	1	1	1	0	0	1	3	0	7	Moderate
Mazzuca et al. (2018)	1	1	1	1	0	1	4	0	9	Moderate
Shachar et al. (2017) [38]	1	1	1	1	1	2	4	1	12	High
Shachar et al. (2017) [39]	1	1	1	1	0	2	3	1	10	High
Thanestada et al. (2022)	1	1	1	2	1	2	4	1	13	High
Ueno et al. (2020)	1	1	1	1	1	0	4	1	10	High
Van Den Berg et al. (2018)	1	1	1	1	0	2	3	0	9	Moderate
Wong et al. (2014)	1	1	1	1	0	2	4	1	11	High

#### Study characteristics

Characteristics of the 11 included articles are summarized in Table 2. Included studies reviewed the relationship between body composition and chemotherapy toxicities or adverse events occurring in individuals undergoing treatment for breast cancer. They included a total of 2881 women, with study numbers ranging from 21 to 1395 participants. Six of the studies reported a mean age with a range from 49 to 52.8 [39, 41–43, 45, 47], and the additional five studies reported a median age with a range from 51.8 to 56 [17, 38, 40, 44, 46].Eight studies investigated women with stage I–III histologically confirmed breast cancer [17, 39–43, 45, 46], two examined metastatic breast cancer [44, 47], and the remaining study did not document cancer staging [38]. Body composition was assessed using abdominal CT scans at the level of the third lumbar vertebrae in nine of the studies [17, 38, 39, 41–45, 47], and the remaining two used BIA (*n*=1) [40] and DEXA (*n*=1) [46].

**Table 2:** Summary of included studies investigating body composition and chemotherapy toxicities

*Author*	*Population*	*Study design*	*Mean/median age*	*Number of participants*	*Body composition*	*Toxicity comparison*	*Outcome/findings*
Aleixo et al. (2021) [43] USA	Women Early-stage breast cancer (stage I–III) Treated with (neo)adjuvant chemotherapy	Retrospective	Mean age=51 (range 24–82)	*n*=338	CT images SMD	Relative risk for an adverse event during chemotherapy including hospitalization, dose reduction, early treatment discontinuation	In multivariable analysis (adjusted dichotomized), patients with myosteatosis (low SMD) in all 3 groups had a significantly higher risk for any adverse event (*p*=0.003; 0.001; 0.01) and a significantly higher risk for dose reduction (*p*= 0.04; 0.05; 0.03). For the psoas group, there was a significant risk of early treatment discontinuation (p=0.03)
Aleixo et al. (2020) [39] USA	Women ≥21 Stage I–III breast cancer Treated with (neo) adjuvant chemotherapy	Retrospective cohort	Mean age=51 (SD 11.6)	*n*= 338	CT images SMI SMD Sarcopenia	Risk of adverse events including hospitalization, dose reduction, early treatment discontinuation	Multivariable analysis showed myosteatosis (SMD) at all 3 thresholds resulted in a significantly higher risk for any adverse event (unadjusted) (*p*=0.0004; 0.002; <0.0001). Higher age and comorbidities were significant independent predictors of adverse events (*p*≤0.05). Lower BSA was significantly associated with dose reduction (*p*=0.03). SMI and VAT were not found to be independently significant for any reported toxicities.
Cespedes et al. (2019) [41] USA	Women Stage II–III breast cancer Treated with anthracycline (AC) and taxane-based chemotherapy	Retrospective	Mean age 52.8 (SD:10.2)	*n*=1395	CT images	Risk of low RDI (<0.85), hospitalization during chemotherapy, and hematological toxicities	Greater visceral and intramuscular adiposity increased the odds of not receiving the full dose (<0.85) of chemotherapy (OR 1.19; 1.16), and hospitalization (OR 1.17; 1.09) during chemotherapy. All forms of adiposity increased the odds of ceasing chemotherapy early (OR 1.16). Greater muscle mass decreased the odds of hematologic toxicities (OR 0.84).
Gouérant et al. (2013) [42] France	Women early breast cancer Obese (BMI≥30) and Non-obese (BMI<30) Treated with (neo)adjuvant chemotherapy	Retrospective	Mean age Obese 52.8 Non-obese 52.0	*n*=200 (100 obese, 100 non-obese) Those with CT scan Obese *n*=50 Non-obese *n*=39	BMI and if available CT images fat mass fat-free mass	Relative dose intensity, dose reduction, delayed chemotherapy, changes in regime and chemotherapy disruptions Unplanned hospitalization	FEC100 chemotherapy regimen: Grade III–IV neutropenia was significantly more frequent in non-obese patients (*p*=0.009). No difference between obese and non-obese groups for febrile neutropenia (*p*=0.28). Docetaxel chemotherapy regimen: Low RDI was significantly more frequent in obese patient (*p*=0.008) usually caused by mucositis or cutaneous toxicities Fat mass was the only factor predictive of docetaxel RDI reduction (*p*=0.004).
Mazzuca et al. (2018) [17] Italy	Stage I–III histologically confirmed breast cancer At least 4 cycles of AC-based adjuvant chemotherapy	Retrospective	Median age 54 (range 39–72)	*n*=21	CT images Sarcopenia BMI BSA	Hematologic toxicities Neurotoxicities gastrointestinal toxicities	Multivariate analysis showed SMI to be the only independent predictor of severe toxicity (G3-4) (*p*=0.0282).
Shachar et al. (2017) [44] USA	Women ≥21 years metastatic breast cancer Taxane-containing chemotherapy	Retrospective	Median age 55 (range 34–80)	*n*=40	CT scans BSA BMI LMD SMD SMG	Hematologic toxicities Neurotoxicities Gastrointestinal toxicities Dose reductions Treatment delays Hospitalization due to chemotherapy toxicity and death	Sarcopenic participants experienced significantly more grade 3–4 toxicities compared with non-sarcopenic (*p*=0.02). Patients who developed grade 3–4 toxicity during cycles 1–3 had significantly lower SMG (*p*=0.04) and lower SMD (*p*=0.01). BMI, BSA, and estimated LBM was not associated with cycle 1–3 toxicity. Hospitalization was significantly associated with low LBM (*p*=0.03), low SMD (*p*=0.03), and low SMG (*p*=0.01). Sarcopenia was significantly associated with any adverse event (*p*=0.06).
Shachar, et al. (2017) [45] USA	Women >21 years Early breast cancer (stage I–III) anthracycline and taxane-based (neo)adjuvant chemotherapy	Retrospective	Mean age 49 (range 23–57)	*n*=151	CT imaging BSA BMI LMD SMD SMG	Hematologic toxicities Neurotoxicities gastrointestinal toxicities Dose reductions Treatment delays Hospitalization due to chemotherapy toxicity and death	Low SMG (<1475) significantly increased the risk of hematologic toxicity (*p*=0.03). Low SMG resulted in twice the hospitalization risk (*p*=0.05) A 5 HU decrease in SMD resulted in a 19% increased relative risk of hospitalization.
Thanestada et al. (2022) [40] Thailand	Women ≥21 years Stage I–III breast cancer anthracycline (AC) and/or taxane-based chemotherapy	Prospective observational	Median age 56 (range 26–75)	*n*=144	BIA FM FFM bone mass PA BMI: (weight (kg)/[height (m)]^2^ FFMI: (fat-free mass (kg)/[height (m)]^2^ )	Neutropenia-related adverse events RDI	Multivariant analysis found low FFMI only to be the independent predictor of CAEs (*p*=0.001).
Ueno et al. (2020) [38] Japan	Women ≥18 Breast cancer (neo)adjuvant epirubicin plus cyclophosphamide (EC) chemotherapy	Retrospective	Median age 54 (IQR 44.3–66)	*n*=82 Sarcopenia *n*=10 non-sarcopenia *N*=53	CT imaging SMA and SMD Visceral adipose tissue Sarcopenia (SMI < 40cm^2^/m^2^ Height, weight, and BMI	LAE	Participants who experienced severe LAEs had a significantly lower baseline SMI (*p*=0.013). Sarcopenic participants were significantly more likely to have a severe LAE (*p*=0.004). No significant difference between other variables.
van den Berg et al. (2018) [46] Netherlands	Women ≥18 Stage I–IIIB breast cancer (neo)adjuvant chemotherapy.	Retrospective	Median age 51.8 (IQR 46.8;59.1)	*n*=172	DEXA Body weight (kg) fat mass (kg relative to TBW) lean mass (kg relative to TBW). Appendicular skeletal mass, SMI 4 groups were then created 1: NLM and NFM 2:NLM and HFM 3:LLM and NFM 4:LLM and HFM	Treatment modifications chemotherapy dose reductions, cycle delays, premature cessation of treatment change in chemotherapy regimen due to toxicities	Higher BMI associated with higher risk ofTIM (*p*<0.01). Higher absolute and relative fat mass associated with higher risk of TIM (*p*=0.01; <0.01). Higher percentage of relative lean mass associated with lower risk of TIM (*p*<0.01). Absolute lean mass not associated with treatment modifications (*p*=0.76). Group 4 had higher TIM than group 1 (HR: 1.33).
Wong et al. (2014) [47] Singapore	Asian women ≥21 advanced/metastatic breast cancer Neo-adjuvant chemotherapy	Prospective open-label phase II	Mean age 50.4 (SD 10.1)	*n*=50 (study 1-arm A)) *n*=50 (study 2) Only *n*=84 In final analysis	CT images BMI BSA Total and intra-abdominal fat measurement (in cm^3^) with fat ratio defined as the ratio of intra-abdominal to total abdominal fat volume.	Hematologic toxicities	Intra-abdominal fat volume and fat ratio were significantly higher in participants who developed grade 3 and 4 leukopenia (*p*=0.014; 0.012) but were not significant for grade 3 and 4 neutropenia (*p*=0.139; 0.120).

*CT* computed tomography, *BIA* bioelectrical impedance analysis, *BMI* body mass index, *BSA* body surface area, *CAE* composite adverse event, *DEXA* duel-energy x-ray absorptiometry, *FFMI* fat-free mass index, *HFM* high-fat mass, *HR* hazard ratio, *IQR* interquartile range, *LAE* laboratory adverse event, *LLM* low lean mass, *LMD* low muscle density, *NFM* normal fat mass, *NLM* normal lean mass, *OR* odds ratio, *PA* phase angle, *RDI* relative dose intensity, *SD* standard deviation, *SMD* skeletal muscle density, *SMG* skeletal muscle gauge, *SMI* skeletal muscle index, *TIM* toxicity-induced treatment modification, *VAT* visceral adipose tissue

In the studies included in this review, different chemotherapy regimens were evaluated including anthracycline (AC) and/or taxane-based chemotherapy regimen (*n*=4) [40–42, 45], AC only (*n*=2) [38, 47], and taxane-based (*n*=1) [44]. For the remaining studies, the chemotherapy regimens were not documented (*n*=4) [17, 39, 43, 46].

Data collected on toxicities or severe adverse events included early treatment cessation, dose delays, and reduced relative dose intensity (RDI) (*n*=4) [39, 42, 43, 46], severe adverse events grade 3 or higher according to the CTCAE (*n*=2) [17, 47], or both (*n*=5) [38, 40, 41, 44, 45].

Due to the large variation in study designs, methods of measurement, and outcomes, quantitative analysis was not considered to be appropriate; therefore, results have been presented in a narrative form.

### Methods for assessing body composition

Of the 11 studies included in this review, nine used CT scans [17, 38, 39, 41–45, 47], one used DEXA [46], and one BIA [40] to evaluate body composition. All studies using CT for analysis assessed scans at the level of the third lumbar vertebra. Data obtained from CT scans were analyzed using validated Hounsfield units (HU) ranges to differentiate between tissue types. Although all studies utilizing CT scans for body composition analysis identified fat mass and muscle mass, different combinations of and calculations using these parameters and their effect on chemotherapy were analyzed. Four studies examined the effect of the skeletal muscle index (SMI), a ratio of skeletal muscle area and height [17, 38, 44, 45]. SMI is often used as a diagnostic tool for sarcopenia; however, it does not consider muscle strength. Two studies investigated the effect of myosteatosis or low skeletal muscle density due to intramuscular adiposity on chemotherapy toxicities [39, 43]. The study by Aleixo et al. (2020) investigated three different thresholds that had been observed in previous literature to indicate myosteatosis [39]. Both studies by Schachar et al. explored the impact of the skeletal muscle gauge (SMG), calculated by multiplying SMI by SMD, as both factors have been shown in previous studies to be predictive of chemotherapy toxicities [44, 45]. The three remaining studies focused their analysis on the effect of adiposity, both visceral and intramuscular on chemotherapy toxicities [41, 42, 47].

The final two studies used BIA (*n*=1) [40] and DEXA (*n*=1) [46] for body composition analysis. The study by Thanestada et al. (2022), using BIA, investigated FM, FFM (muscle mass and bone mass), and phase angle which reflects the cell membranes. This method of analysis is not able to assess skeletal muscle density as in CT evaluation. The BIA device used by many of the participants in this study was a consumer-level product, due to difficulty with the availability of a clinical BIA assessment device, with only 74 of the total 148 participants being measured with both clinical and consumer-level devices. For participants measured with the two devices, a linear correlation analysis was completed and showed a significant correlation between the FFM results [40]. In the final study by van den Berg et al. [46], body composition was evaluated by trained technicians using DEXA scans with fat mass and lean mass calculated. The participants from this study were divided into four different groups according to their body composition. All individual body composition parameters were assessed along with hazard ratios (HRs) being reported for each of the four groups.

### Classification of sarcopenia

In this review, six studies explored sarcopenia and its impact on chemotherapy toxicities [17, 38–40, 44, 45]. As in previous literature, studies included in this review used different thresholds to indicate sarcopenia, one using ≤38.5cm^2^/m^2^ [17], one ≤40cm^2^/m^2^ [38], and two ≤41cm^2^/m^2^ [44, 45],and one study by Aleixo (2020) analyzed three separate thresholds of ≤38.5cm^2^/m^2^, <40cm^2^/m^2^, and <41cm^2^/m^2^. Of these studies, Schachar et al. (2017) examined sarcopenic obesity using thresholds of ≤41cm^2^/m^2^ in conjunction with a BMI ≥30kg/m^2^ [45]. Additionally, Thanestada et al. (2022) using BIA for body composition assessment reported on a fat-free mass index (FFMI) that was calculated by dividing FFM (kg) by height in m^2^, with <11.4kg/m^2^ documented as the threshold to indicate sarcopenia.

### Methods for assessing chemotherapy toxicities or adverse events

Four of the studies included in this review recorded dose reduction or delay, early treatment cessation, or hospital admission, due to chemotherapy toxicities as adverse events [39, 42, 43, 46]. All four of these studies were retrospective. A further five studies used both reduced RDI, or hospitalization, and graded toxicities to signify an adverse event due to chemotherapy [38, 40, 41, 44, 45].

The two additional studies documented an adverse event as toxicities graded 3 or greater using the CTCAE grading scale without reference to treatment delay, dose reduction, or hospitalization [17, 47].

Nine of the 11 included studies were retrospective with toxicity data being extracted from electronic medical records, which for some studies limited the data available. In the study by Ueno et al. [38], only laboratory adverse events were included due to other toxicities possibly being omitted in the medical records. Of the remaining six studies examining graded toxicities, three investigated hematologic toxicities only [40, 41, 47], a further two hematologic and gastrointestinal toxicities [44, 45], and the final study by Mazzuca et al. which was one of only two prospective studies reported all toxicities recorded due to chemotherapy. Only two of the 11 included studies were prospective in nature allowing the design of the study to pre-determine the classification of adverse events [40, 47].

### The effect of body composition on chemotherapy toxicities

#### Stage I–III breast cancer

Eight of the 11 included studies investigated women with histologically confirmed stage I–III breast cancer [17, 39–43, 45, 46]. Five of these studies found a significant association between skeletal muscle parameters and chemotherapy toxicities [17, 39, 40, 43, 45]. Both studies by Aleixo et al. examined the effect of myosteatosis or low SMD, SMI, and high visceral adipose tissue (VAT) on chemotherapy toxicities. Toxicities included dose reduction, early cessation of treatment, and hospitalization due to toxicities. In multivariate analyses, Aleixo et al. (2020) demonstrated myosteatosis (using 3 different thresholds; SMD < 41 HU, <37.8 HU, <32.5 HU) resulted in a significantly higher risk of any adverse event (unadjusted *p*=0.0004; 0.002; <0.0001). At thresholds of < 41 cm^2^/m^2^ and < 40 cm^2^/m^2^, SMI was found to be significantly associated with decreased RDI (*p*=0.04; 0.01); however, it was not found to be independently predictive at any threshold for the reported toxicities. VAT >100 cm^2^ was significantly associated with early treatment discontinuation (*p*=0.02); however, it was not independently predictive. The other study by Aleixo et al. (2021) found a significant association between myosteatosis (calculated by both SpiceOMatic and Picture Archiving and Communication Systems (PACS) analyses), using thresholds of <32.5HU for SpiceOMatic, <47.5HU for PACS psoas, and <35.5HU for PACS erector spinae, and any adverse event (adjusted dichotomized *p*=0.003; 0.001; 0.03 respectively). The authors also reported a significant association between dose reduction for all three assessment thresholds (*p*=0.04; 0.05; 0.03). Only the PACS psoas (<47.5HU) was significant for early treatment discontinuation and none were significant for increased hospitalization (adjusted dichotomized *p*=0.06; 0.09; 0.07).

Of the four additional studies examining the effect of sarcopenia (indicated by low SMI or in one study low FFMI) on adverse events during chemotherapy for individuals with stage I–III breast cancer [17, 39, 40, 45], three showed SMI to be statistically predictive of some or all toxicities [17, 40, 45]. Schachar et al. (2017) investigated a different body composition parameter (SMG) and found participants with low SMG were twice as likely to suffer any toxicities than those with high SMG as well as having twice the risk of hospitalization (*p*=0.05). They were also statistically more likely to experience grade 3 or higher hematologic toxicities (*p*=0.03). Additionally, this study demonstrated SMG and LBM were the most reliable predictors of grade 3–4 toxicities [45].

Thanestada et al. (2022) used BIA for body composition analysis to explore the association between fat-free mass and composite adverse events (CAEs), including grade 4 neutropenia, febrile neutropenia, and relative dose intensity of less than 85%, and reported that low FFMI (<14.85kg/m^2^) was the only independent predictor of CAEs during chemotherapy treatment (*p*<0.001). It should be noted that using the international consensus threshold of <11.4kg/m^2^ for sarcopenia, none of the participants in this study were identified as being sarcopenic at their baseline measure [48].

For the three remaining studies, analyses focused on fat or adipose tissue factors and their association with chemotherapy toxicities [41, 42, 46]. Gouérant et al. (2013) examined the difference between obese (BMI ≥30kg/m^2^) and non-obese participants undergoing treatment with docetaxel. The authors reported that obese patients have significantly more chance of reduced RDI (*p*=0.008); however, there was no difference between the two groups for hematologic toxicities. In a separate multivariate analysis, FM was also found to be a significant independent predictor of reduced RDI (*p*=0.004) [42]. An observational cohort study with 1395 participants investigated adiposity and its relationship to hematologic toxicities, RDI (<15% or more), hospitalizations, treatment delay, and early cessation of treatment. Both visceral and intra-muscular (IM) adiposity was found to put a patient at increased risk of low RDI (visceral OR=1.19, IM OR=1.16), hospitalization (visceral OR=1.17, IM OR=1.09), and completing chemotherapy early (visceral OR=1.16, IM OR=1.14) while a greater muscle mass was shown to reduce the risk of hematologic toxicities (OR=0.84) [41].

The final study by van den Berg  et al. [46] investigated fat mass and lean mass in relation to body weight using DEXA for body composition analysis. Both absolute and relative high-fat mass were associated with an increased risk of treatment modifications due to toxicities (HR per 5kg increase absolute fat mass =1.14; HR per 5% increase relative fat mass =1.21). Modifications included dose reductions or delays, changes in the treatment regimen, and early cessation of treatment. Absolute lean mass did not affect treatment; however, a higher relative lean mass reduced the risk of treatment modifications [46].

#### Metastatic breast cancer

Two studies examined the effect of body composition on chemotherapy toxicities in women with advanced or stage IV metastatic breast cancer [44, 47]. Wong et al. (2014) reported on Chinese and Malay participants and investigated body fat and muscle volume, and their effect on pharmacokinetics and hematologic toxicities. The authors observed that high intra-abdominal fat volume even in underweight individuals was significantly associated with grade 4 leukopenia (underweight *p*=0.027; overweight *p*=0.034) [47]. The second study by Shachar et al. (2017) examining chemotherapy toxicities in individuals with advanced or metastatic breast cancer calculated SMG and noted low SMG was predictive of grade 3–4 toxicities during cycles 1 to 3 (*p*= 0.04), and hospitalization (*p*=0.01), and was borderline significant for any adverse event (*p*=0.06). Additionally, SMD was associated with grade 3–4 toxicities during cycles 1 to 3 (*p*=0.01) and hospitalizations (*p*=0.03). This study used an SMI of ≤41cm^2^/m^2^ as the threshold for sarcopenia. Individuals considered sarcopenic were significantly more likely to experience any adverse event including dose reduction or delay, hospitalization, or grade 3–4 toxicity (hematological and gastrological) (*p*=0.02). Although biological agents were used in conjunction with chemotherapy in this study, these were not thought to be a causative factor for toxicities [44].

For the final study by Ueno et al. [38], it was unclear as to the cancer staging of participants. This study which used the threshold for sarcopenia as <40cm^2^/m^2^ reported that sarcopenic participants were significantly more likely to suffer grade 3 or higher laboratory adverse events than non-sarcopenic participants (*p*=0.004), with no other body composition parameters demonstrating any significant association [38].

## Discussion

Chemotherapy remains a conventional treatment for breast cancer; however, BSA, the method still commonly used to calculate dosage for individuals, dates back to the 1950s [12, 14, 49]. Most of the literature to support this approach came from either retrospective studies or inter-species analyses on drug toxicities [12]. The theory behind using BSA to calculate dose is that the larger the individual, the better their ability to metabolize chemo toxic drugs due to a greater area for drug distribution [14]. However, as BSA only considers the individuals’ height and weight, it does not factor differences in body composition which can often be considerable, even in individuals with similar BSA [50]. As cytotoxic drugs administered for the treatment of cancer have a narrow therapeutic index, overdosing can lead to increased toxicities, and under-dosing may result in a reduced therapeutic effect [9, 10, 12, 15]. The aim of this systematic review was to examine the effect of body composition on chemotherapy toxicities and its potential role in personalizing chemotherapy dosage for individuals with breast cancer. Following an extensive literature review, 11 studies were identified that met the inclusion criteria for this review. Although all included studies showed specific body composition components to have significant associations with chemotherapy toxicities, the actual components identified varied between studies, in addition to different thresholds being reported for sarcopenia and myosteatosis.

Although all studies included in this review assessed body composition parameters other than simply BMI or BSA, various assessment methods for analysis were used. CT scans at the level of the third lumbar vertebra were used in most of the included studies (82%) [17, 38, 39, 41–45, 47].The other two studies used BIA (*n*=1) [40] and DEXA (*n=*1) [46] to analyze body composition. Unlike CT, DEXA and BIA are unable to distinguish intra-muscular adiposity and muscle quality; however, the value of BIA or more recently BIS as a tool for assessing body composition is that it is non-invasive and does not subject the individual to radiation, allowing them to be monitored throughout their treatment. As secondary sarcopenia has been linked to cancer [51], the ability to prospectively monitor any changes over time could be beneficial to physicians to individualize treatment during both planning, and throughout chemotherapy treatment.

CT has the advantage of being able to distinguish between skeletal muscle as well as subcutaneous, visceral, and intra-muscular adiposity, allowing the calculation of SMD (determined by the extent of adipose tissue within the muscle) [34]. Eight of the nine included studies using CT scans to assess body composition were retrospective studies [17, 38, 39, 41–45] . This resulted in participants included in the studies being limited to those who had acceptable CT scans within a proposed time frame, which varied from 45 days to 6 months prior to chemotherapy. Additionally, it may have resulted in bias, as individuals who had CT staging scans were liable to have more advanced disease and therefore are potentially more likely to be sarcopenic, as secondary sarcopenia has been reported to be associated with cancer and other chronic diseases [51].

Five of the included studies analyzed SMD either individually and/or as part of an additional calculation SMG [38, 39, 43–45]. These parameters are only able to be assessed using CT, with low SMD indicating myosteatosis. With four of the five studies reporting SMD to be associated with adverse events or chemotherapy toxicities including dose reduction or delay, early treatment discontinuation, grade 3–4 toxicities, and hospitalization [39, 43–45], this indicates the potential value of CT as an important means of assessing body composition for individuals being treated with chemotherapy. With CT scans often being performed as a diagnostic tool to assess cancer staging prior to treatment, it would only require extra calculations, with no further exposure to radiation to assess body composition. Although the remaining study by Ueno et al. [38] did not find a significant association between SMD and laboratory adverse events, this study did not investigate toxicities such as reduced RDI, treatment delays, or hospitalizations.

Of the two studies by Shachar et al. examining the significance of the SMG (calculated by multiplying SMI by SMD to incorporate both muscle quantity and quality), one reported SMG to be the best independent predictor of grade 3–4 toxicities in individuals with early breast cancer [44], with the other finding SMG to be a significant factor along with sarcopenia in predicting grade 3–4 toxicities in individuals with metastatic breast cancer [45]. This further justifies the value of CT imaging as an assessment tool for body composition if individuals are undergoing cancer-staging CT scans, as neither BIA nor DEXA can assess intramuscular adiposity.

One of the issues when investigating the effect of body composition on adverse events or severe toxicities during chemotherapy treatment is the lack of standardized thresholds to indicate sarcopenia. More recently, the impact of sarcopenia on both prognosis and toxicities experienced during treatment for various cancer groups has been investigated, with some studies reporting decreased overall survival and increased chemotherapy toxicities [26, 51–54]. CT scans at the level of L3 level are currently the gold standard for calculating SMI, which can then be used to diagnose sarcopenia [55]. Low SMI is considered a prognostic factor in diagnosis; however, there are various classifications in the literature as to what constitutes sarcopenia and there is no consensus as to the threshold at which this diagnosis should be made, with previously identified thresholds in women ranging from 29.6 to 41 cm^2^/m^2^, and in men from 36 to 55.4 cm^2^/m^2^ [56]. Furthermore, there are various definitions of sarcopenia with many also factoring muscle strength and function in addition to low muscle mass [51], which is not reflected in SMI. Although five studies included in this review examining the effect of sarcopenia, diagnosed by low SMI or FFMI, found it to be significantly associated with increased chemotherapy toxicities [17, 38, 40, 44, 45], with only one reporting no significance in multivariable analyses [43], none of these studies assessed muscle function or strength, and based their diagnosis of sarcopenia purely on low SMI. Additionally, the thresholds used to determine sarcopenia ranged from ≤38.5 to <41cm^2^/m^2^. As there is no consensus as to the definition of sarcopenia, evaluating its effect on chemotherapy toxicities can be complicated as the diagnostic criteria may differ between studies.

Although there are common chemotherapy drugs utilized for the treatment of breast cancer, various regimens may be used, depending on factors such as tumor pathology and cancer staging, among others. Unsurprisingly, the studies included in this review reflected this with several regimens being investigated. Different chemotherapy regimens can result in various toxicities [57, 58], and combinations of chemotherapy drugs such as anthracycline and taxane, though generally resulting in improved clinical response, are often associated with increased toxicities [57]. Additionally, treatment for cancer is multi-modal and is tailored to individual needs. Granulocyte colony-stimulating factor (G-CSF) and other associated biological agents are commonly used in conjunction with chemotherapy [59, 60], making it difficult to ascertain whether toxicities are the result of chemotherapy or another treatment intervention. G-CSF has been shown to reduce the risk of neutropenia [61]. Shachar et al. (2017) reported that 37.5% of participants received a biological agent in addition to chemotherapy [44].

The variations in chemotherapy regimens and additional interventions in conjunction with chemotherapy all contribute to difficulty comparing studies examining the role of body composition on chemotherapy toxicities. Furthermore, toxicities collected predominantly included data on planned versus delivered chemotherapy and hospitalization with graded toxicities reported in only seven of the included studies likely due to limited data available in the medical records [17, 38, 40, 41, 44, 45, 47].

Although these studies show promising results supporting the role of body composition as a means of predicting chemotherapy toxicities, there were limitations in this review. Sample sizes ranged from small to moderate reducing their statistical power. Additionally, comparisons between studies were difficult given the variations in chemotherapy regimens and concurrent treatments. Moreover, with nine of the 11 studies being retrospective, the complexity is compounded as it can be challenging to extract relevant data from EMRs and draw firm conclusions. A lack of consensus on thresholds in the literature for both sarcopenia and myosteatosis may also result in biases depending on which threshold is applied.

## Conclusion

The findings from this review suggest that body composition analysis prior to the commencement of chemotherapy for breast cancer may be a valuable tool to predict toxicities; however, future prospective studies allowing for greater control over clinical parameters are indicated to generate robust data going forward. As toxicities due to chemotherapy may result in individuals receiving a reduced therapeutic dose, potentially affecting prognosis, this is an area that requires further investigation as a better understanding of the effect of different body composition parameters on chemotherapy toxicities may allow interventions to be put in place to mitigate this risk. Furthermore, improved knowledge in this area may give rise to a more reliable way of individualizing chemotherapy dosage.

## Supplementary information

ESM 1 (PDF 13 kb)

ESM 2 (PDF 122 kb)

## Data Availability

All data generated for this review are included in the manuscript and/or the supplementary files.
