# Supplementary material for: Body composition and chemotherapy toxicities in breast cancer: a systematic review of the literature
Source: J Cancer Surviv. 2024 Jan 11;19(3):914–29. doi: 10.1007/s11764-023-01512-z (PMC12081505; doi:10.1007/s11764-023-01512-z)
Supplement: Supplementary file 1 — (PDF 13 kb) [file 11764_2023_1512_MOESM1_ESM.pdf]

Journal of Cancer Survivorship

Body composition and chemotherapy toxicities in breast cancer: A systematic review of the literature

Lori Lewis[1], Belinda Thompson[1], Rhiannon Stellmaker[1], Louise Koelmeyer[1]

[1] Australian Lymphoedema Education, Research & Treatment (ALERT) Program, Department of Health Sciences, Faculty of Medicine, Health and Human Sciences, Macquarie University, Sydney, NSW, Australia

Supplementary File 1: Search Strategy:

Database	Date	Search Strategy
Embase	2012 - 10th August 2022	1. breast cancer<br>2. chemotherapy<br>3. cytotoxic OR toxicit* OR side effect*<br>4. Body composition OR sarcopenia OR muscle mass OR fat mass OR malnutrition OR undernutrition OR sarcopenic obesity OR anorexia<br>5. filter 3: Humans, Full text, English
PubMed	2012 - 10th August 2022	1. breast cancer<br>2. chemotherapy<br>3. cytotoxic OR toxicit* OR side effect*<br>4. Body composition OR sarcopenia OR muscle mass OR fat mass OR malnutrition OR undernutrition OR sarcopenic obesity OR anorexia<br>5. filter 3: Humans, Full text, English
Cochrane Central Register of Controlled Trials	2012 - 10th August 2022	1. breast cancer<br>2. chemotherapy<br>3. cytotoxic OR toxicit* OR side effect*<br>4. Body composition OR sarcopenia OR muscle mass OR fat mass OR malnutrition OR undernutrition OR sarcopenic obesity OR anorexia<br>5. filter 3: Humans, Full text, English
CINAHL	2012 - 10th August 2022	6. breast cancer<br>7. chemotherapy<br>8. cytotoxic OR toxicit* OR side effect*<br>9. Body composition OR sarcopenia OR muscle mass OR fat mass OR malnutrition OR undernutrition OR sarcopenic obesity OR anorexia<br>filter 3: Humans, Full text, English
