# Supplementary material for: Body composition and chemotherapy toxicities in breast cancer: a systematic review of the literature
Source: J Cancer Surviv. 2024 Jan 11;19(3):914–29. doi: 10.1007/s11764-023-01512-z (PMC12081505; doi:10.1007/s11764-023-01512-z)
Supplement: Supplementary file 2 — (PDF 122 kb) [file 11764_2023_1512_MOESM2_ESM.pdf]

Journal of Cancer Survivorship

Body composition and chemotherapy toxicities in breast cancer: A systematic review of the literature

Lori Lewis[1], Belinda Thompson[1], Rhiannon Stellmaker[1], Louise Koelmeyer[1]

[1] Australian Lymphoedema Education, Research & Treatment (ALERT) Program, Department of Health Sciences, Faculty of Medicine, Health and Human Sciences, Macquarie University, Sydney, NSW, Australia

Supplementary file 2: Articles excluded after full-text review

Author(s)	Title	Grounds for Exclusion
**Abdollahi, et al. 2019**	The Effect of Dietary Intervention Along with Nutritional Education on Reducing the Gastrointestinal Side Effects Caused by Chemotherapy Among Women with Breast Cancer Nutrition and cancer, 71(6), 922-930	Unable to isolate the effect of body composition
**Adams, et al. 2016**	Impact of resistance and aerobic exercise on sarcopenia and dynapenia in breast cancer patients receiving adjuvant chemotherapy: a multicenter randomized controlled trial. Breast Cancer Research and Treatment, 158(3), 497-507	Unable to isolate the effect of body composition and not assessing chemotherapy toxicities
**Aleixo, et al. 2020**	Association of body composition with function in women with early breast cancer. Breast Cancer Res Treat, 181(2), 411-421	Chemotherapy toxicities are not discussed only physical function and quality of life from PRO's
**Courneya, et al. 2013**	Effects of exercise dose and type during breast cancer chemotherapy: multicenter randomized J Natl Cancer Inst, 105(23), 1821-1832	Body composition parameters and on-treatment chemotherapy toxicities are not clear. More focussed on physical function and long-term toxicities such as peripheral neuropathy
**Courneya, et al. 2014**	Subgroup effects in a randomised trial of different types and doses of exercise during breast cancer chemotherapy. Br J Cancer, 111(9), 1718-1725	Body composition is used as an outcome measure, not chemotherapy toxicities
**De Souza, et al. 2021**	Nutritional Intervention Contributes to the Improvement of Symptoms Related to Quality of Life in Breast Cancer Patients Undergoing Neoadjuvant Chemotherapy: a Randomized Clinical Trial. Nutrients, 13(2), 589-589	Body composition not analyzed
**Delrieu, et al. 2021**	Sarcopenia and serum biomarkers of oxidative stress after a 6-month physical activity intervention in women with metastatic breast cancer: results from the ABLE feasibility trial. Breast Cancer Res Treat, 188(3), 601-613	Exercise is the intervention

Author(s)	Title	Grounds for Exclusion
**Durkin, et al. 2022**	Body composition and chemotherapy toxicity in women with early breast cancer (CANDO-3): protocol for an observational cohort study. BMJ open, 12(2)	Protocol only for an observational study
**Ghoreishi, et al 2016**	Association of age and body mass index with paclitaxel-induced peripheral neuropathy in patients with breast cancer [Journal: Conference Abstract]. Annals of Oncology, 27	Abstract Only
**Grupińska, et al 2021**	Beneficial Effects of Oral Nutritional Supplements on Body Composition and Biochemical Parameters in Women with Breast Cancer Undergoing Postoperative Chemotherapy: A Propensity Score Matching Analysis. Nutrients, 13(10)	The comparison was nutritional supplements and outcome measures were body composition and biochemical parameters, not chemotherapy toxicities
**Hertz, et al 2022**	Muscle mass affects paclitaxel systemic exposure and may inform personalized paclitaxel dosing. Br J Clin Pharmacol, 88(7), 3222-3229	systemic paclitaxel concentrations, or pharmacokinetics (PK), and infusion duration are used for comparison
**Lee, et al 2019**	Sarcopenia as a predicting factor for chemotherapy in advanced breast cancer patients. European Journal of Surgical Oncology, 45(2)	Abstract only
**Mazzuca, et al 2018**	Effect of nutritional support with highly purified, whey proteins for malnutrition and sarcopenia in patients affected with stage IIIII colorectal or breast cancer: A blind, placebo controlled, randomized clinical trial [Conference Abstract]. Journal of Clinical Oncology. Conference, 36(15 Supplement 1)	The primary objective is the nutritional status between two arms, PROLYOTIN and placebo, abstract only
**Porciúncula, et al 2013**	The influence of body composition on quality of life of patients with breast cancer. Nutr Hosp, 28(5), 1475-1482	QoL used as outcome measure not chemotherapy toxicities
**Sabel, et al 2012**	Sarcopenia to predict tolerance of adjuvant breast cancer chemotherapy [Conference Abstract]. Journal of Clinical Oncology. Conference, 30(27 SUPPL. 1)	Abstract only
